# Levels and sources of polycyclic aromatic hydrocarbons (PAHs) near hospitals and schools using leaves and barks of *Sambucus nigra* and *Acacia melanoxylon*

**DOI:** 10.1007/s10653-023-01825-z

**Published:** 2024-01-16

**Authors:** Katiuska Alexandrino, Nazly E. Sánchez, Fausto Viteri

**Affiliations:** 1 Grupo de Investigación en Biodiversidad, Medio Ambiente y Salud (BIOMAS), Facultad de Ingenierías y Ciencias Aplicadas, Universidad de Las Américas, Vía a Nayón, Quito, 170124 Ecuador; 2 Departamento de Ingeniería Ambiental y Sanitaria, Universidad del Cauca, 190007 Popayan, Colombia; 3 Grupo de Protección Ambiental (GPA), Facultad de Ciencias de La Ingeniería e Industrias, Universidad UTE, Quito, 170527 Ecuador

**Keywords:** Biomonitor, Polycyclic aromatic hydrocarbons, HPLC, Emissions sources, Tree species

## Abstract

Polycyclic aromatic hydrocarbons (PAHs) are among the most studied organic compounds in urban environments, due to their known threat to human health. This study extends the current knowledge regarding the ability of different vegetative parts of different tree species to accumulate PAHs. Moreover, exposure intensity to PAHs in areas frequented by population susceptible to adverse health effects of air pollution is evaluated. For this, leaves and barks of *Sambucus nigra* (*S. nigra*) and *Acacia melanoxylon* (*A. melanoxylon*) were collected at urban areas in the Andean city of Quito, at seven points near hospitals and schools. A methodology, previously developed, for the extraction, purification, and quantification of PAHs associated with the leaves and bark of *S. nigra* was employed and also validated for leaves and bark of *A. melanoxylon*. The total PAH level varied from 119.65 ng g^−1^ DW (dry weight) to 1969.98 ng g^−1^ DW (dry weight) with naphthalene (Naph), fluoranthene (Flt), pyrene (Pyr), chrysene (Chry), and benzo[a]pyrene (BaP) predominating in all samples. The results indicate that the leaves and bark of tree species studied have certain abilities to bio-accumulate PAH according to their molecular weight. The leaves of *S. nigra* and bark of *A. melanoxylon* showed the highest ability to accumulate PAHs, mainly those with high and medium molecular weight, respectively. The highest incidence of light molecular weight PAHs was found in the leaves of *A. melanoxylon*. Furthermore, coal combustion, biomass burning, and vehicle emissions were identified as the main PAHs sources. Concentrations of PAHs associated with tree species suggest an affectation in areas frequented by populations susceptible to air pollution. This fact shows the importance of regulatory scheme to significantly improve the air quality in the city integrating a knowledge-based decision-making.

**Supplementary Information:**

The online version contains supplementary material available at 10.1007/s10653-023-01825-z.

## Introduction

Polycyclic aromatic hydrocarbons (PAHs) are important harmful pollutants to the environment and health (Hou et al., 2018; Perera et al., 2014); thus, there is a great interest in addressing the public health risk they cause, especially in urban areas frequented by populations susceptible to the adverse health effects of air pollution, with concentration diagnostics in the atmosphere and cost-effective action by means of the identification of pollutant sources.

PAHs are found in the gas phase or associated with particulate phase which contributes to their ubiquity in the environment. In general, low molecular weight PAHs (2- to 3-rings, LMW) are frequently found in the gas phase, medium molecular weight PAHs (4-rings, MMW) are generally present in both gas and particulate phase, and high molecular weight PAHs (5- and 6-rings, HMW) are mainly distributed in particulate phase (Dat & Chang, 2017). Common sources of PAHs in urban areas mainly include pyrogenic (or pyrolytic, e.g., vehicle emissions, typically associated with high molecular PAHs) and petrogenic (petroleum, e.g., crude oil, bitumen, asphalt, typically associated with low molecular PAHs) sources (Hwang et al., 2003; Ratola et al., 2011a).

In the last decades, the use of plants for biomonitoring of urban air pollution, including PAHs, has gained importance due to its easy access and low cost (De Nicola, et al., 2016; Prajapati & Tripathi, 2008; Sari et al., 2021). PAHs in the gas phase can be absorbed by stomatal uptake and/or they may diffuse through the wax layer and the cuticular membrane, while particle-bound PAHs are accumulated on the leaf surface due to the high interaction with the lipophilic waxy cuticle layer (De Nicola et al., 2016; Holoubek et al., 2000; Lehndorff & Schwark, 2004).

Several studies have shown the successful use of leaves of trees to measure the concentration of PAHs in an area (Alfani et al., 2001; Bakker et al., 2000; Fellet et al., 2016; van Drooge et al., 2014). Bark of trees has also shown good capacity to accumulate PAHs, due to its high lipid content, and porous and almost inert surface (Niu et al., 2019; Pereira et al., 2019; Ratola et al., 2009), although research with this type of matrix is scarcer. Accumulation of PAHs on vegetative parts is plant species dependent, mainly to the chemical, morphological and physiological characteristics of each tree species (Fellet et al., 2016; Pereira et al., 2019; Rodriguez et al., 2012). The vegetative parts of a same tree species have also shown to have different abilities to accumulate PAHs (Ratola et al., 2009, 2012; Yin et al., 2011). Identification of tree species, and different vegetative parts of a same tree species, with the best ability to accumulate air pollutants is still poorly known and constitutes an important research topic, mainly because data can allow to choose the most appropriate for a specific purpose and can also help to identify which factors control the PAH accumulation.

*Sambucus nigra* (*S. nigra*, tree or shrub of the family Adoxaceae) and *Acacia melanoxylon* (*A. melanoxylon,* tree of the family Fabaceae) are two widely distributed tree species in the Andean areas of Latin America. However, studies on their use as biomonitors are still very scarce. Although the literature shows evidence on the use of some vegetative parts of these two tree species as biomonitors of some air pollutants, such as heavy metals (Armijos et al., 2022; Kolodziej et al., 2012; Topolska et al., 2020), to our knowledge, there is no information to date on the evaluation of the potential use of their leaves and barks for PAHs. Thus, the present work aims to analyze the concentration of PAHs in leaf and bark samples of *S. nigra* and *A. melanoxylon* collected near Hospitals and Schools in the Andean city of Quito, Ecuador. In this way, this work addresses the existing gap by comparing the PAH incidence between different vegetative parts of a same tree species, and between tree species. Moreover, the levels of PAHs in areas frequented by populations susceptible to adverse health effects of air pollution are evaluated which could serve to assess the exposure intensity and to analyze the major emission sources and their contribution.

## Materials and method

The methodology used was previously developed for leaf and bark samples of *S. nigra* (Viteri et al., 2023). In the present work, the performance of such methodology was also evaluated for leaf and bark samples of *A. melanoxylon* showing satisfactory results, as it will be seen later. A brief description of the methodology (sampling procedure, sample pretreatment and the quantitative determination of PAHs) is given below.

### Sampling sites and sampling procedure

**Fig. 1:**
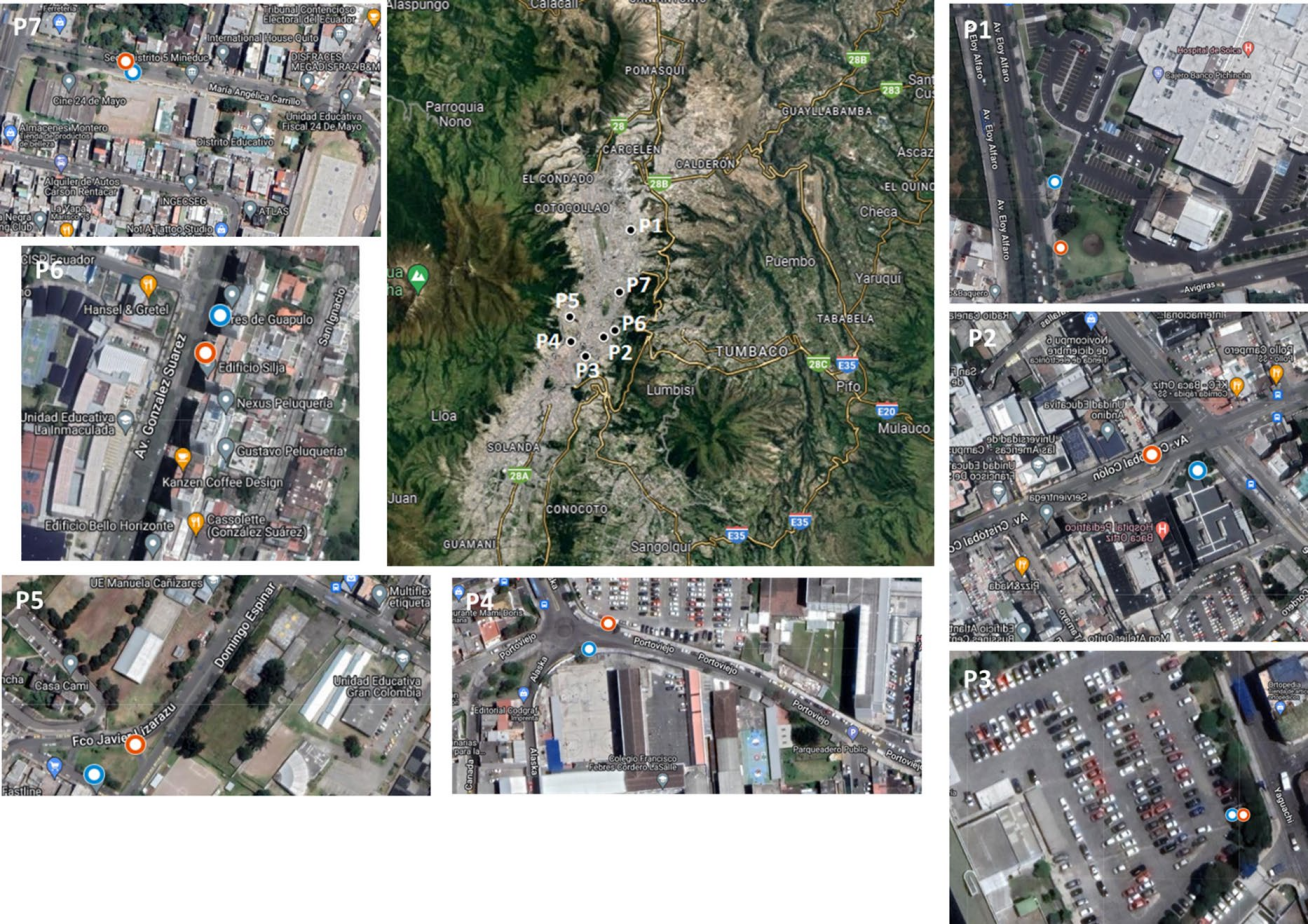
Sampling points in the city of Quito, Ecuador. *Blue* points: *S. Nigra*; *Red* points: *A. melanoxylon*

Tree leaf and bark samples were collected in urban areas of the city of Quito (2850 m.a.s.l.) at seven points (P1-P7) close to hospitals and schools that had both tree species at a maximum distance of 10 m from each other (Fig. 1): P1—Solca Hospital; P2—Baca Ortiz Pediatric Hospital; P3—Eugenio Espejo Hospital; P4—Carlos Andrade Marín Hospital and Francisco Febres Cordero LaSalle School; P5—Gran Colombia and Manuela Cañizares Schools; P6—La Inmaculada School; P7—24 de Mayo School. All the sampling points have a high flow of motor vehicles and some of them with food cooking services in the vicinity.

The sampling was carried out in May 2021. Briefly, a pruning shear was used to collect the leaves from all directions and the outer part of the trees at a height of about 2 m above the ground. For bark, a pre-cleaned stainless-steel knife was used to remove the external layer from all directions of the tree at the height of 1.5 m above the ground. Leaves with evidence of chlorosis or necrosis, as well as barks with moss and lichen on the surface, were avoided. A new pair of powder-free vinyl gloves for each sample was used to avoid cross-contamination.

Leaf and bark samples were placed in ziplock bags, labeled with sample information, wrapped in aluminum to protect from light and refrigerated in a cooler for transport to the laboratory. One fraction of the samples was used to measure the water content, while the other part was stored at -20^◦^C before sample treatment and analysis. The water content was measured by drying, in duplicate, 2 g of leaves and barks for each tree species and site, until constant weight in an oven at 70 ± 2^◦^C (Armijos et al., 2022).

### Sample pretreatment (extraction and clean-up)

Prior to extraction, the samples were defrosted in a desiccator. Then, 2 g of leaves or barks was placed into a baker with 20 mL of a dichloromethane/hexane (1:1 v/v) mixture and immersed in a 420-W ultrasonic bath for 10 min. This procedure was repeated two more times, using fresh solvent mixture, for a total of 30 min of extraction and 60 mL of dichloromethane/hexane mixture. The three extracts were combined into the same round-bottom flask and evaporated up to approximately 1 mL in a Buchi rotary evaporator at 30 °C.

For clean-up of concentrated extract, each sample was transferred onto a Sep-Pak Alumina cartridge (6 cc, 1 g. Waters) previously conditioned with 10 mL of the dichloromethane/hexane mixture. The target analytes were eluted with 10 mL of the dichloromethane/hexane mixture and then with 5 mL of dichloromethane. After cleaning-up, the extracts were again evaporated by rotary evaporator to approximately 1 mL and concentrated using a vacuum concentrator (Genevac miVac) at 40 °C for 30 min. Finally, the samples were reconstituted in 1 mL of acetonitrile, filtered using PVDF syringe filters (32 mm, 0.22 μm) and transferred to 2 mL amber glass vial for subsequent high-performance liquid chromatography (HPLC) analysis.

### Analysis of polycyclic aromatic hydrocarbons

In the present work, 14 PAHs were analyzed (see Table S1 for their structural formulas), namely naphthalene (Naph), acenaphthylene (Acy), phenanthrene (Phen), anthracene (Ant), fluoranthene (Flt), pyrene (Pyr), benzo[a]anthracene (BaA), chrysene (Chry), benzo[b]fluoranthene (BbF), benzo[k])fluoranthene (BkF), benzo[a]pyrene (BaP), dibenzo[a,h]anthracene (DahA), benzo[g,h,i]perylene (BghiP) and indeno[1,2,3-cd]pyrene (IcdP). The quantitative analysis of these PAHs was carried out by a HPLC (Agilent 1260 system) equipped with a ZORBAX Eclipse PAH column (4.6 × 50 nm, 3.5 µm) and an UV detector (Agilent 1260 DAD G4212B) set to wavelengths (λ) of 220 nm, 230 nm and 254 nm. Analytes were separated by gradient elution with acetonitrile (A) and water (B) as mobile phase, at a flow rate of 1.4 mL/min, the injection volume set to 20 μL and the column temperature at 20 °C. The elution program was defined as follows: 0–6 min isocratic 40:60 (v/v) A:B; 6–9.5 min linear gradient from 40 to 100% of A and 9.5–12 min isocratic 40:60 (v/v) A:B. Peak identification and integration were performed by external standard method with the ChemStation software (Agilent Technologies).

### Quality assurance/quality control (QA/QC)

The details about the parameters of the analytical method (linearity, limit of detection (LOD), limit of quantification (LOQ), repeatability and recovery) can be found in Viteri et al. (2023). In brief, the linearity range was from 2.5 to 2500 μg L^−1^ with coefficient of determination (R^2^) greater than 0.9993. The LOD and LOQ values of individual PAHs were in the range of 0.20–13.7 μg L^−1^ and 0.6–41.5 μg L^−1^, respectively. Repeatability, studied as percent relative standard deviation (%RSD), ranged from 0.006 to 4.6% indicating good instrumental precision. Recovery results were in the 64.8–106.4% range for *S. nigra* and in the 48.2–91.3% range for *A. melanoxylon (*see Table 1*).*

**Table 1:** Percentage of recovery (%R) for leaves and barks of *S. nigra* and *A. melanoxylon*

PAH	*S. nigra*			*A. melanoxylon*	
	Leaves		Bark	Leaves	Bark
Naphthalene (Naph)	74.8	56.7		59.8	51.9
Acenaphthylene (Acy)	64.8	58.6		66.5	27.6
Phenanthrene (Phen)	106.4	100.6		73.7	54.5
Anthracene (Ant)	88.9	92.4		87.3	68.3
Fluoranthene (Flt)	90.2	79.9		91.3	64.8
Pyrene (Pyr)	77.4	69.8		65.1	48.2
Benzo[a]anthracene (BaA)	85.1	86.7		73.6	49.6
Chrysene (Chry)	95.7	82.1		91.0	64.2
Benzo[b]fluoranthene (BbF)	72.1	75.7		86.7	69.8
Benzo[k]fluoranthene (BkF)	70.0	73.2		50.2	59.2
Benzo[a]pyrene (BaP)	91.2	69.3		81.7	52.4
Dibenzo[a,h]anthracene (DahA)	83.9	74.7		61.4	61.4
Benzo[g,h,i]perylene (BghiP)	76.4	82.8		76.3	55.7
Indeno[1,2,3-cd]pyrene (IcdP)	75.7	64.3		48.2	37.4

To determine the possible background contamination during the treatment of the sample, including laboratory tools interference, procedural blanks (extraction and clean-up of reagents without vegetative material) were also analyzed. The concentration of PAHs in the samples was corrected based on the procedural blanks whenever they were above the respective LOD (Bakker et al., 2000; Birgül et al., 2011; Busso et al., 2018; Navarro-Ortega et al., 2012; Silva et al., 2015) and recoveries (Bakker et al., 2000; Tham et al., 2008; van Drooge et al., 2014). Moreover, to examine cross-contamination and interference in the HPLC and to check the accuracy of the determination of PAHs, blanks (only acetonitrile) and the standard solution at 100 μg L^−1^ were ran every ten samples. PAH concentrations in all the blank analyses were below the LOD.

### Principal component analysis

Principal component analysis (PCA) has been widely used in biomonitoring studies of air pollution (Alexandrino et al., 2022; Alfani et al., 2001; Fellet et al.,; 2016) in which the pollutants in a same component may indicate a common source. In this work, PCA for each matrix type and tree species was performed to identify the emissions sources using the R software through the R commander interface and the Multivariate Exploratory Data Analysis and Data Mining-FactoMineR package.

## Results and discussion

PAHs were identified in leaf and bark samples of *S. nigra* and *A. melanoxylon* collected at seven sites in urban areas of Quito near Hospitals and Schools. In this section, first, the total and individual concentration of the 14 PAHs in the leaf and bark samples of both tree species is discussed. Subsequently, a comparison of the PAHs levels between both vegetative parts and between tree species is presented. Finally, PAHs sources incidence and their contribution are identified.

### Total and individual PAH concentration in the leaf and bark samples of both tree species

**Fig. 2:**
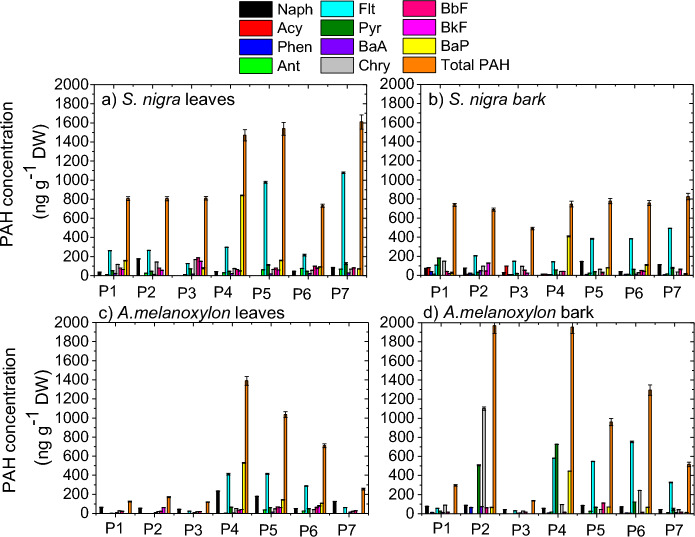
Total and individual PAH concentration in leaves and barks of *S. nigra* (**a** and **b**, respectively) and *A. melanoxylon* (**c** and **d**, respectively) at each site studied

The total and individual PAH concentration in leaves and barks of *S. nigra* and *A. melanoxylon* at each site is plotted in Fig. 2. Total PAH refers to the sum of individual PAH $({\sum }_{1}^{n:14}{PAHs}_{n} )$ found at each site studied. It is observed that the total PAH concentration ranges from 119.65 ng g^−1^ DW to 1969.98 ng g^−1^ DW which is within the same order of magnitude as the results found in the literature for different tree species (Alfani et al., 2001; Augusto et al., 2010; Holoubek et al., 2000; Navarro-Ortega et al., 2012; Niu et al., 2019; van Drooge et al., 2014; Wu et al., 2019; Wu et al., 2019; Yang et al., 2007).

It should be noted that DahA, BghiP and IcdP were not detected in the samples, as has also been reported in other works (Fernández et al., 2011; Ratola et al., 2011a; Fellet et al., 2016Orecchio et al., 2008). This could be because heavy PAHs, mainly particulate-bound compounds, are very likely to remain on the surface of leaves and barks rather than diffusing and accumulating in the inner compartment, becoming more prone to suffer the effect of external environmental factors such as heavy rain and wind (Amigo et al., 2011; Busso et al., 2018; Guidotti et al., 2003; Navarro-Ortega et al., 2012; Piccardo et al., 2005; Tomashuk et al., 2012; Wang et al., 2009).

**Fig. 3:**
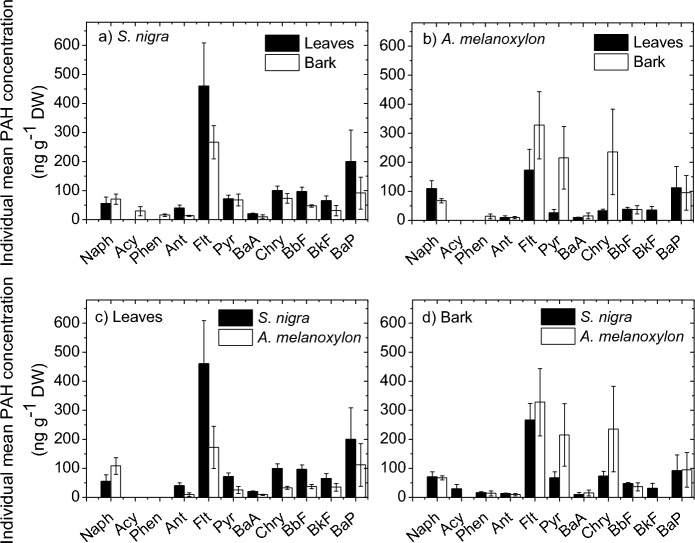
Individual mean PAH profiles for leaves and barks of *S. nigra* and *A. melanoxylon*

To help to figure out which PAHs are the most concentrated in the samples, Fig. 3 shows the individual mean PAH profiles for leaves and barks of *S. nigra* and *A. melanoxylon*. Individual mean PAH refers to the mean of each individual PAH found at all sites studied in each matrix and tree species. Among all PAHs studied, Naph, Flt, Pyr, Chry and BaP show the highest concentrations. This result agrees with those observed in other works (Alfani et al., 2001; Busso et al., 2018; Ratola et al., 2009; Rodriguez et al., 2010), except for BaP. This PAH is the usual marker for carcinogenic levels of PAHs in environmental studies and its high levels could indicate a high human exposure risk (IARC, 2013). The high incidence of BaP could be attributed to gasoline exhaust that is known to contribute for more BaP emissions than diesel engines (Wu et al., 2012).

### Differences between leaf and bark samples in PAH concentration

It is observed from Fig. 2a and 2b that the total PAH concentration in leaves of *S. nigra* is, in general, higher than that in bark. A *t* test (95% confidence interval) confirmed a statistical difference. This is in line with the result obtained by Ratola et al. (2009) in their work using needles and barks of *Pinus pinaster* Ait. and *Pinus pinea* L. On the other hand, in general, for *A. melanoxylon*, the total PAH concentration is higher in bark than in leaves (Fig. 2c and 2d), this being mainly due to the higher concentration of the 4-ring PAHs Flt, Pyr and Chry in bark (Fig. 3b), although a non-statistically significant difference is observed.

Due to the scarcity of comparative studies in the literature between the capacity of leaf and bark of a same tree species to accumulate PAH, a further comparison is not possible. However, studies reporting the potential of leaves and bark to accumulate metals also show contradictory results. For example, Dogan et al. (2010) indicated that the accumulation of metals in the bark of *Pinus brutia* was higher than that in needles. The same was observed by Sawidis et al. (2011), using needles and barks of *Platanus orientalis* and *Pinus nigra*. However, Solgi et al. (2020) indicated that levels of metals in leaves of *Fraxinus excelsior* and *Pinus eldarica* were higher than those in bark. Thus, the distribution of pollutants in different vegetative parts of a same tree seems to be species dependent. In this regard, further research is required to determine the ability of different vegetative parts to retain PAHs depending on the tree species.

**Fig. 4:**
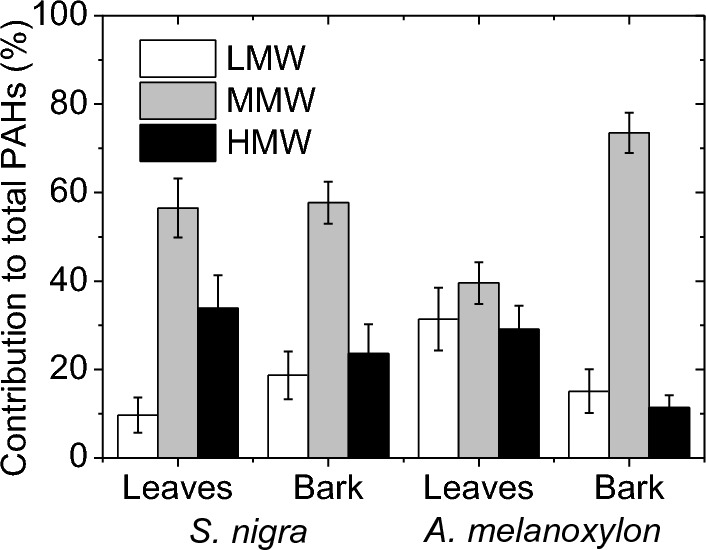
Aromatic ring patterns of PAHs for leaves and barks of *S. nigra* and *A. melanoxylon*

The patterns of PAH incidence in the leaves and barks of both tree species are shown in Fig. 4, which indicates the distribution according to the molecular weight classification (contribution (%) of LMW, MMW, HMW to total PAH).

It is observed from Fig. 4 that, for the two tree species, 4-ring PAHs (MMW) are the dominant in both sample matrices, with a similar percentage in the leaves and bark of *S. nigra* (56.5 and 57.7%, respectively), and being almost double in the bark of *A. melanoxylon* than in the leaves (73.5 and 39.5%, respectively). The high incidence of 4-ring PAHs agrees with that observed in previous biomonitoring studies (Busso et al., 2018). 4-ring PAHs are present in both vapor and particulate matter and are easily undergoing wet and dry deposition enhancing the uptake levels (Amigo et al., 2011; Augusto et al., 2010; Busso et al., 2018). The incidence of LMW PAHs was not as high, mainly in leaves of *S. nigra*, maybe probably due to photodegradation or resuspension into the atmosphere due to their higher vapor pressures (higher volatility) (Amigo et al., 2011; Ratola et al., 2010). On the other hand, the percentage of HMW PAHs was relatively high, and it is mainly due to the high concentration of BaP found in the samples (Figs. 2 and 3).

The leaves and bark of *S. nigra* present the same trend of higher incidence of MMW PAHs, followed by HMW and finally by LMW PAHs, which is in line with that observed by Tian et al. (2019) in leaves of 8 common tree species in Shanghai, China. Moreover, leaves of *S. nigra* collect a higher proportion of HMW PAHs than bark (33.8% vs. 23.6%), and bark captures a higher proportion of LMW PAHs than leaves (18.7% vs. 9.7%). This is opposite to that observed in the work of Ratola et al. (2012) on the use of needles and barks of two pine species (*Pinus pinaster* Ait. and *Pinus pinea* L.) as biomonitors, which showed that LMW PAHs are predominant in needles, while HMW PAHs are predominant in bark. Moreover, Fig. 4 also shows that, although the leaves and bark of *A. melanoxylon* have the same trend of higher incidence of MMW PAHs, followed by LMW and finally by HMW PAHs, leaves of *A. melanoxylon* tend to accumulate more LMW and HMW PAHs than bark. The way leaves and bark of each tree species interact with the surrounding air could be an important factor controlling the PAH uptake. Some leaves and barks better retain particulate matter, while others interact mainly with the vapor phase, and this also has to do with the morphological, physiological and chemical properties of the vegetative parts of the tree species.

### Differences between tree species in PAH concentration

It is clear that *S. nigra* and *A. melanoxylon* have different abilities to capture PAH. Specifically, a *t* test (95% confidence interval) indicates a statistically significant difference in the total concentration of PAHs between leaves of both tree species, with the mean concentration of individual PAHs being higher in the leaves of *S. nigra* than that in the leaves of *A. melanoxylon,* except for Naph (Fig. 3c). On the other hand, a non-statistically significant difference in the total concentration of PAHs between bark of both tree species is found. However, the mean concentrations of Pyr and Chry are found to be three times higher in the bark of *A. melanoxylon* than in *S. nigra* (Fig. 3d).

It is evident from Fig. 4 that leaves of *S. nigra* are more prone to capture MMW and HMW PAHs than those of *A. melanoxylon*, whereas leaves of *A. melanoxylon* are more prone to capture LMW PAHs. Regarding the bark samples, higher percentage of both LMW and HMW PAHs is observed in *S. nigra* than in *A. melanoxylon*, while higher percentage of MMW PAHs is observed in *A. melanoxylon* than in *S. nigra*. Differences between tree species toward PAHs were previously reported (Amigo et al., 2011; Baldantoni et al., 2014; Fellet et al., 2016; Navarro-Ortega, 2012; Piccardo et al., 2005; Ratola et al., 2010; Ratola et al., 2011b; Ratola et al., 2012; Rodriguez et al., 2012). Morphological, physiological and chemical characteristics, including stomatal density, specific area, surface roughness and lipid content, could be a reason for the differences in the distribution between tree species, as they have been identified to play an important role in the accumulation of air pollutants, including PAHs (Baldantoni et al., 2014; Franzaring & Eerden, 2000; Moeckel et al., 2008; Simonich & Hites, 1995; Wu et al., 2019; Yang et al., 2008). In fact, some studies indicate that, depending on the nature of the PAH, its accumulation will depend on one characteristics or another. For example, Tian et al. (2019) evaluated the effects of different leaf characteristics on their ability to accumulate PAHs and found that LMW PAHs were mainly affected by leaf morphological characteristics, whereas MMW and HMW PAHs were mainly affected by wax content. Moreover, Yang et al. (2008) found that specific surface area and stomata density affect the levels of HMW PAHs and lipid content the levels of LMW and MMW PAHs.

The experimental study of the characteristics of leaves and bark, including stomatal density, specific leaf area and lipid content, of *S. nigra* and *A. melanoxylon* is not considered in this work. However, values of some of these characteristics for these two tree species are reported in the literature. For example, the stomatal density on the abaxial surface of leaves of *S. nigra* was reported to be in the range of 71–139.32 mm^2^ (Amini et al., 2021; Atkinson & Atkinson, 1958), while that of *A. melanoxylon* was reported to be in the range of 306 – 473 mm^2^ (Scarr, 2011). Stomatal density has a high significant effect on the uptake rate of PAHs (Tao & Hornbuckle, 2001) and has been found to be positively (Fellet et al., 2016) and negatively (Tian et al., 2019), correlated with the content of PAHs. In the present work, the higher the stomatal density of leaves the lower the total PAH concentration, which is consistent with that observed by Tian et al. (2019). However, the leaves of *A. melanoxylon* are more prone to capture LMW PAHs than those of *S. nigra* (Fig. 4). This could be because light PAHs can penetrate more readily into leaf inner tissues through the stomata than heavy PAHs and then can be accumulated in higher proportions (Huang et al., 2018).

Future works should be focused on studying the morphological, physiological and chemical characteristics of the leaves and barks of *S. nigra* and *A. melanoxylon* in order to be able to identify a more accurate correlation between the concentration of PAHs and these characteristics and then to define those that most control the uptake and accumulation of PAHs in each vegetative part and tree species.

### Analysis of sites and possible sources of PAHs

**Fig. 5:**
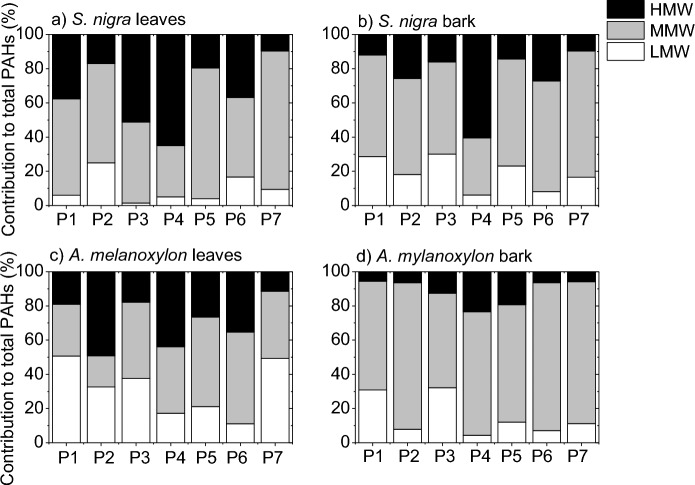
Aromatic ring patterns of PAHs for each site studied for leaves and barks of *S. nigra* (**a** and **b**) and *A. melanoxylon* (**c** and **d**)

Difference between sites in total PAH concentration is observed from Fig. 2, which is expected given the main sources of atmospheric PAHs. Both leaves and bark of *S. nigra* indicate that sites P4, P5 and P7 have the highest total PAH levels, while both leaves and bark of *A. melanoxylon* indicate site P4 as being the one with the highest total PAH level. In this way, Fig. 5 shows the PAHs patterns for each site studied for leaves and barks of the two tree species. It is evident that, in general, at sites P5 and P7, 4-ring PAHs dominate, and it is due mainly to the high concentration of Flt that contributes largely to the total PAH concentration (Fig. 2). Fluoranthene is associated with pyrogenic activities, such as coal combustion and biomass burning (Pulster et al., 2019). In fact, in site 5 there are some soccer fields that make this place crowded by many people and for this reason it is normal to find street vendors preparing food on the street (using gas or coal) to sell. This could be contributing to PAH emissions, mainly pyrogenic PAHs, in that area. Also, at site 7, there is the presence of numerous restaurants than can also be contributing to the high presence of Flt in that area. Regarding site P4, in general, HMW PAHs dominate (Fig. 5). High contribution of HMW PAHs is often attributed to gasoline exhaust (Pereira et al, 2019). The dominance of HMW PAHs at site P4 is seemed to be due to the high concentration of BaP (Fig. 2), which, as mentioned above, is commonly associated with gasoline exhaust (Wu et al., 2012). At site P4 there is the Hospital Carlos Andrade Marin and Francisco Febres Cordero LaSalle School, which means a large circulation of vehicles in that area, in addition to the fact that the sampling point is next to a roundabout whose presence has been indicated to be relevant for PAH levels (Alexandrino et al., 2022).

To complement these observations, Table 2 and 3, and Figs. S1-S4 show the results for the PCA applied to the PAH concentrations found in the leaf and bark samples of *S. nigra* and *A. melanoxylon* from all the sites studied. The results of PAH concentrations were reduced to three (leaves of *S. nigra* and leaves and bark of *A. melanoxylon*) and four components (bark of *S. nigra*), whose eigenvalues were higher than 1. The components explained between 83.8 and 95.8% of data variability.

**Table 2:** Principal component analysis for leaves (normal) and barks (italic) of *S. nigra.*

	Dim. 1	Dim. 2	Dim. 3	Dim. 4
Naph	0.036	*−*0.557 ***0.860***	**0.798** *0.310*	*0.018*
	*−0.355*			
Acy	*−*	−	−	−
	***0.778***	*−0.0010*	*−0.370*	*0.349*
Phen	−	−	−	−
	***0.856***	*0.376*	*0.166*	*−0.099*
Ant	**0.773**	0.201	0.061	−
	*−0.350*	***0.907***	*0.074*	*−0.171*
Flt	**0.847**	0.384	0.174	−
	***−0.855***	*0.318*	*0.211*	*0.272*
Pyr	**0.632**	**0.682**	0.142	−
	***0.500***	*0.464*	*−0.339*	*0.070*
BaA	**0.711**	0.076	−0.302	−
	*0.185*	*−0.343*	***0.887***	*−0.211*
Chry	**−0.864**	0.118	0.311	−
	***0.912***	*0.362*	*0.059*	*0.023*
BbF	**−0.674**	**0.673**	−0.068	−
	*0.183*	*−0.358*	*0.047*	***0.872***
BkF	**−0.870**	0.374	−0.244	−
	*0.350*	*−0.286*	***0.889***	*−0.047*
BaP	0.121	−0.531	**−0.737**	−
	*−0.223*	*−0.491*	*−0.505*	***−0.627***
Eigenvalue	4.2	1.8	1.5	−
	*3.6*	*2.7*	*2.3*	*1.4*
% of variance	46.6	20.6	16.5	−
	*33.1*	*24.7*	*20.6*	*13.1*
Cumulative % of variance	46.6	67.2	83.8	−
	*33.1*	*57.8*	*78.4*	*91.5*

PAHs with the highest absolute value in each dimension are in bold

**Table 3:** Principal component analysis for leaves (normal) and barks (italic) of *A. melanoxylon*

	Dim. 1	Dim. 2	Dim. 3
Naph	**0.686**	**0.708**	0.133
	*0.521*	*0.431*	***0.603***
Phen	*–*	*−*	*−*
	***0.970***	*0.195*	*−0.110*
Ant	0.772	**−0.468**	0.409
	*−0.561*	**0.711**	0.322
Flt	**0.988**	0.114	0.024
	***−0.601***	***0.614***	*0.076*
Pyr	**0.993**	0.040	−0.089
	*0.340*	***0.729***	*−0.584*
BaA	−0.179	0.485	**0.837**
	***0.980***	*−0.034*	*−0.038*
Chry	**0.974**	0.138	−0.072
	***0.953***	*0.196*	*−0.039*
BbF	**0.835**	−0.429	0.301
	*0.228*	*0.459*	***0.787***
BkF	**0.700**	−0.508	−0.100
	**−**	−	−
BaP	**0.736**	0.530	−0.413
	*−0.196*	***0.771***	*−0.575*
Eigenvalue	5.7	1.7	*1.8*
	*4.0*	*2.5*	1.2
% of variance	63.7	19.0	13.0
	*44.0*	*27.6*	*19.8*
Cumulative % of variance	63.7	82.7	95.8
	*44.0*	*71.6*	*91.4*

PAHs with the highest absolute value in each dimension are in bold

PCA patterns were not completely identical for all vegetative parts and species studied, which can partly be explained by the differences in morphological, physiological and chemical characteristics of the samples and by the sensitivity to processes causing the removal of deposited material (e.g., wash-off, wind abrasion). However, some similarities can be observed, mainly in leaf samples, in where Naph and BaP are in the same dimension suggesting the same emission source for these PAHs. Naph is linked to light vehicular traffic (Guidotti et al., 2003) and is also a component of diesel fuels (Preuss et al., 2003), while BaP is linked to gasoline exhaust (Wu et al., 2012). Moreover, in a previous work (Alexandrino et al., 2022), Naph and BaP were associated with traffic related emissions, specifically acceleration and braking activities, i.e., with the presence of traffic lights, roundabouts, intersections, curves, and speed bumps. Moreover, the PCA results show that, in general, Naph and BaP are related to the Site P4, which supports the above discussion that traffic emissions are pointed out as an important source of emissions in this urban area. On the other hand, in general, 3- and 4-ring PAHs, mainly Flt and Ant, are grouped in the same dimension which may originate from biomass burning, and diesel and natural gas combustion (Dias et al., 2016) and are more related to the Sites P5 and P7. These results indicate coal combustion, biomass burning, and vehicle exhaust as the main sources of PAHs emissions.

The implementation of tree planting as a strategy for ecological restoration in urban environments, particularly in areas with high levels of air pollutants such as PAHs, should take into account several key aspects. Firstly, monitoring of pollutants in urban areas is crucial. This involves identifying hotspots, distribution, types, and levels of pollutants. Our study demonstrates that the efficacy of aromatic remediation is dependent on the species of tree, hence characterization of pollutant presence is a crucial task. Secondly, it is essential to select tree species with the highest capacity for pollutant absorption. In our work, both tree species studied show good performance to accumulate PAHs. However, the selection of the best species and vegetative part depends on the type of PAHs (LMW, MMW, and HMW) that is to be identified, since the results show differences between tree species and vegetative parts to accumulate PAHs types. Thirdly, the planting of trees should be strategically planned in terms of density and height. Research indicates that dense and tall vegetation can significantly reduce pollutants, with some studies (Uribe et al., 2023) suggesting a reduction impact greater than 50%, particularly for vegetation belts approximately 10 m thick near pollution sources. Additionally, factors such as climate, geographical location, and landscaping should be considered in selecting the most suitable species. Avoid invasive species that can disrupt the ecosystem balance.

It is important to remember that tree planting is only one strategy among many for mitigating air pollutants like PAHs. Comprehensive mitigation efforts should include the control of stationary and mobile pollution sources, urban planning that incorporates integrated urban design, efficient monitoring and evaluation systems, and environmental education strategies, among others, to effectively reduce pollutants in the atmosphere.

## Conclusions

This work extends the knowledge about the comparison of the incidence of PAHs between different vegetative parts of a same tree species (leaves and bark), and between tree species (*S. nigra* and *A. melanoxylon*), in areas with presence of vulnerable populations to air pollution. Both tree species studied show good performance as biomonitors for PAHs. However, the selection of the best species and vegetative part depends on the type of PAHs (heavy, medium, and light molecular weight PAHs) that is to be identified, since the results show differences between tree species and vegetative parts to accumulate PAHs types. The total PAH concentration in leaves of *S. nigra* was higher than that in bark due to a higher capacity of leaves to capture high molecular weight PAHs, while the total PAH concentration in bark of *A. melanoxylon* was higher than that in leaves. The 4-ring PAHs, mainly fluoranthene, pyrene, and chrysene, were dominant in all samples, with the highest incidence being in the bark of *A. melanoxylon.* The highest incidence of heavy, medium, and light molecular weight PAHs was found in leaves of *S. nigra*, bark of *A. melanoxylon* and leaves of *A. melanoxylon*, respectively. As chemical, morphological, and physiological characteristics of leaves and bark have been identified in the literature to play an important role in the accumulation of air pollutants, including PAHs, future investigations should be directed to investigate these characteristics of *S. nigra* and *A. melanoxylon*. Affectation by PAHs in areas frequented by populations susceptible to air pollution was observed, with coal combustion, biomass burning, and vehicle exhaust identified as the main sources of PAHs emissions.

### Supplementary Information

Below is the link to the electronic supplementary material.

Supplementary file1 (DOCX 778 kb)
