# Correction: Levels and sources of polycyclic aromatic hydrocarbons (PAHs) near hospitals and schools using leaves and barks of Sambucus nigra and Acacia melanoxylon

**DOI:** 10.1007/s10653-024-01985-6

**Published:** 2024-04-25

**Authors:** Katiuska Alexandrino, Nazly E. Sánchez, Fausto Viteri

**Affiliations:** 1 Grupo de Investigación en Biodiversidad, Medio Ambiente y Salud (BIOMAS), Facultad de Ingenierías y Ciencias Aplicadas, Universidad de Las Américas, Vía a Nayón, Quito, 170124 Ecuador; 2 Departamento de Ingeniería Ambiental y Sanitaria, Universidad del Cauca, 190007 Popayan, Colombia; 3 Grupo de Protección Ambiental (GPA), Facultad de Ciencias de La Ingeniería e Industrias, Universidad UTE, Quito, 170527 Ecuador

**Correction to: Environ Geochem Health (2024) 46:32** 10.1007/s10653-023-01825-z.

In this article Fausto Viteri was incorrectly denoted as the corresponding author. The only corresponding author of the article should be Katiuska Alexandrino.

The original article has been corrected.

