# Supplementary material for: Levels and sources of polycyclic aromatic hydrocarbons (PAHs) near hospitals and schools using leaves and barks of Sambucus nigra and Acacia melanoxylon
Source: Environ Geochem Health. 2024 Jan 16;46(2):32. doi: 10.1007/s10653-023-01825-z (PMC10791842; doi:10.1007/s10653-023-01825-z)
Supplement: Supplementary file 1 — Supplementary file1 (DOCX 778 kb) [file 10653_2023_1825_MOESM1_ESM.docx]

Table S1. List of the 14 PAHs analyzed and their structural formula

| **PAHs** | **Structural Formula** |
| --- | --- |
|  |  |
| Low molecular weight PAH (LMW) | |
| Naphthalene (Naph) |  |
| Acenaphthylene (Acy) |  |
| Phenanthrene (Phen) |  |
| Anthracene (Ant) |  |
| Medium molecular weight PAHs (MMW) | |
| Fluoranthene (Flt) |  |
| Pyrene (Pyr) |  |
| Benzo[a]anthracene (BaA) |  |
| Chrysene (Chry) |  |
| High molecular weight PAHs (HMW) | |
| Benzo[b]ﬂuoranthene (BbF) |  |
| Benzo[k]ﬂuoranthene (BkF) |  |
| Benzo[a]pyrene (BaP) |  |
| Dibenzo[a,h]anthracene (DahA) |  |
| Benzo[g,h,i]perylene (BghiP) |  |
| Indeno[1,2,3-cd]pyrene (IcdP) |  |


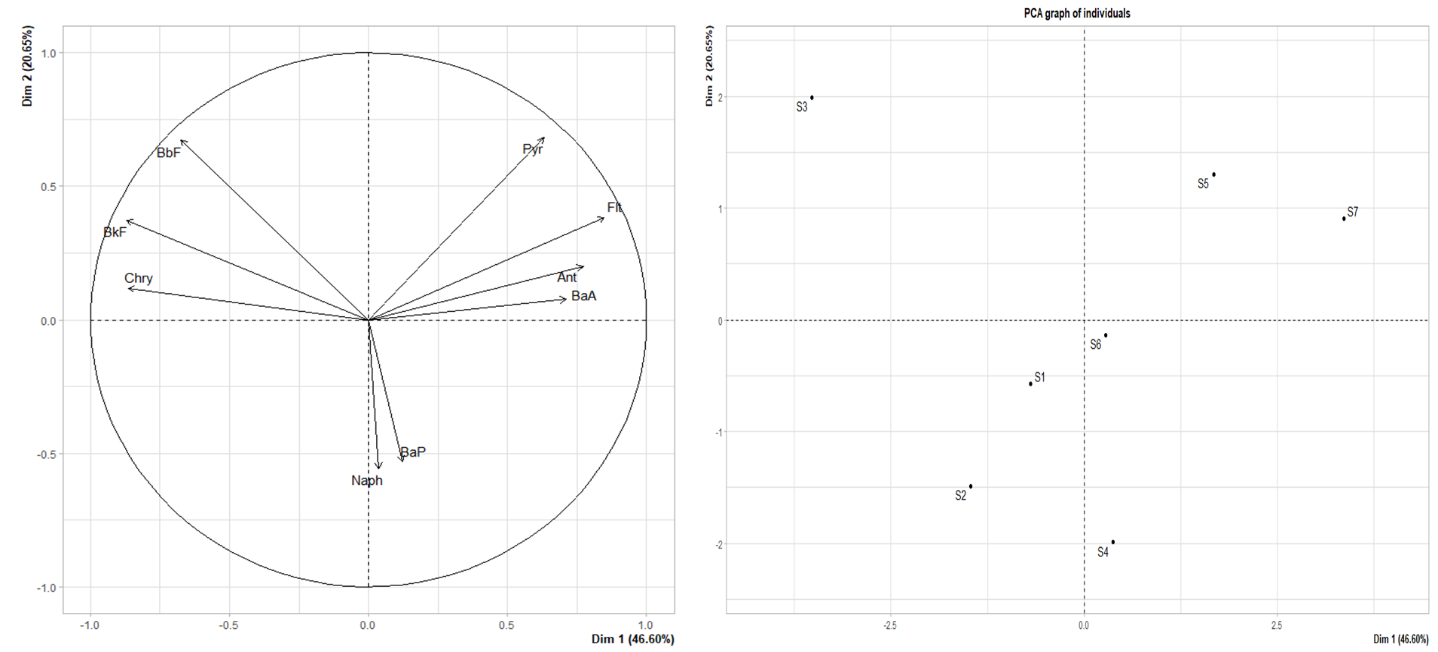


Fig. S1. Principal component analysis based on the two principal components of the data from *Sambucus nigra* leaves.


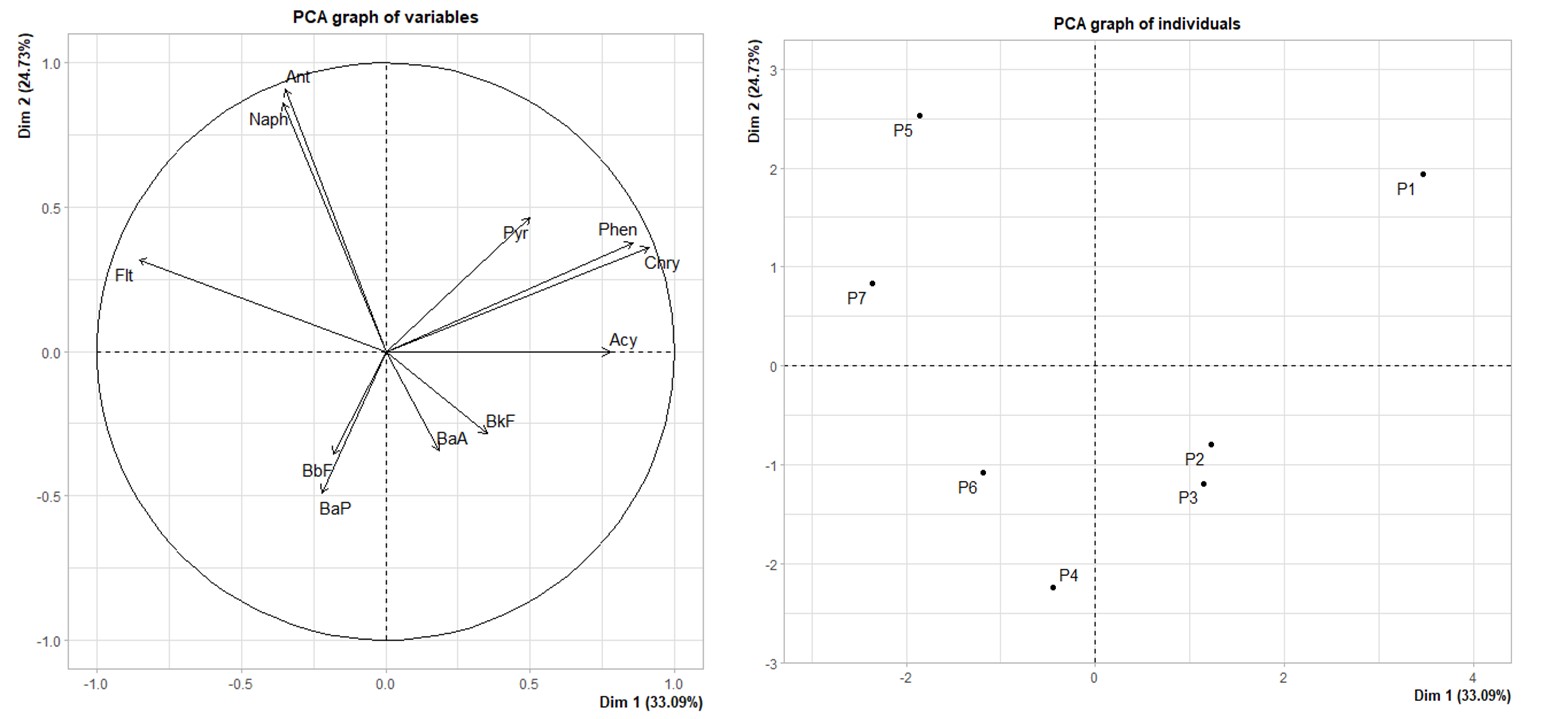


Fig. S2. Principal component analysis based on the two principal components of the data from *Sambucus nigra* bark.


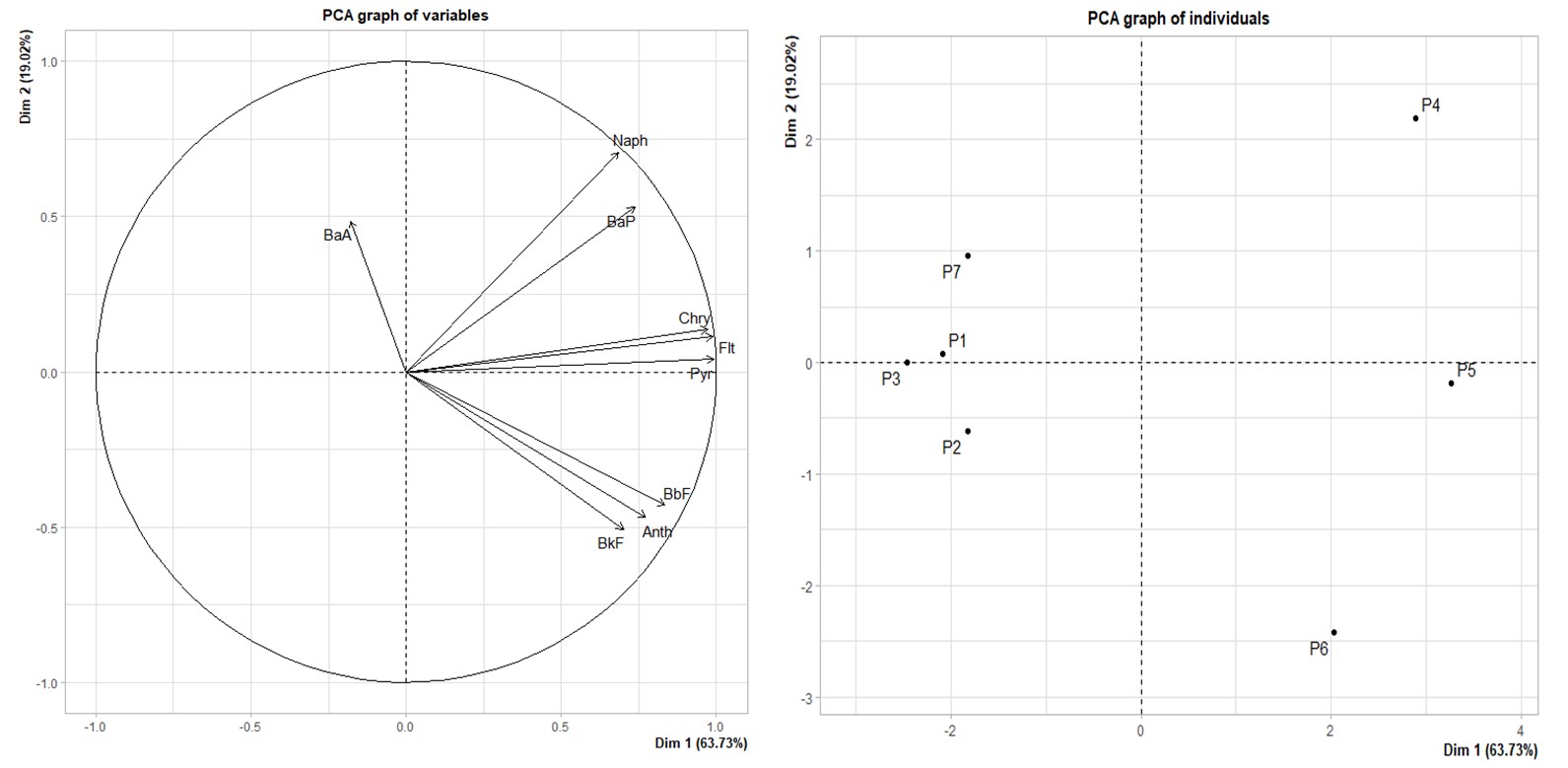


Fig. S3. Principal component analysis based on the two principal components of the data from *Acacia melanoxylon* leaves.


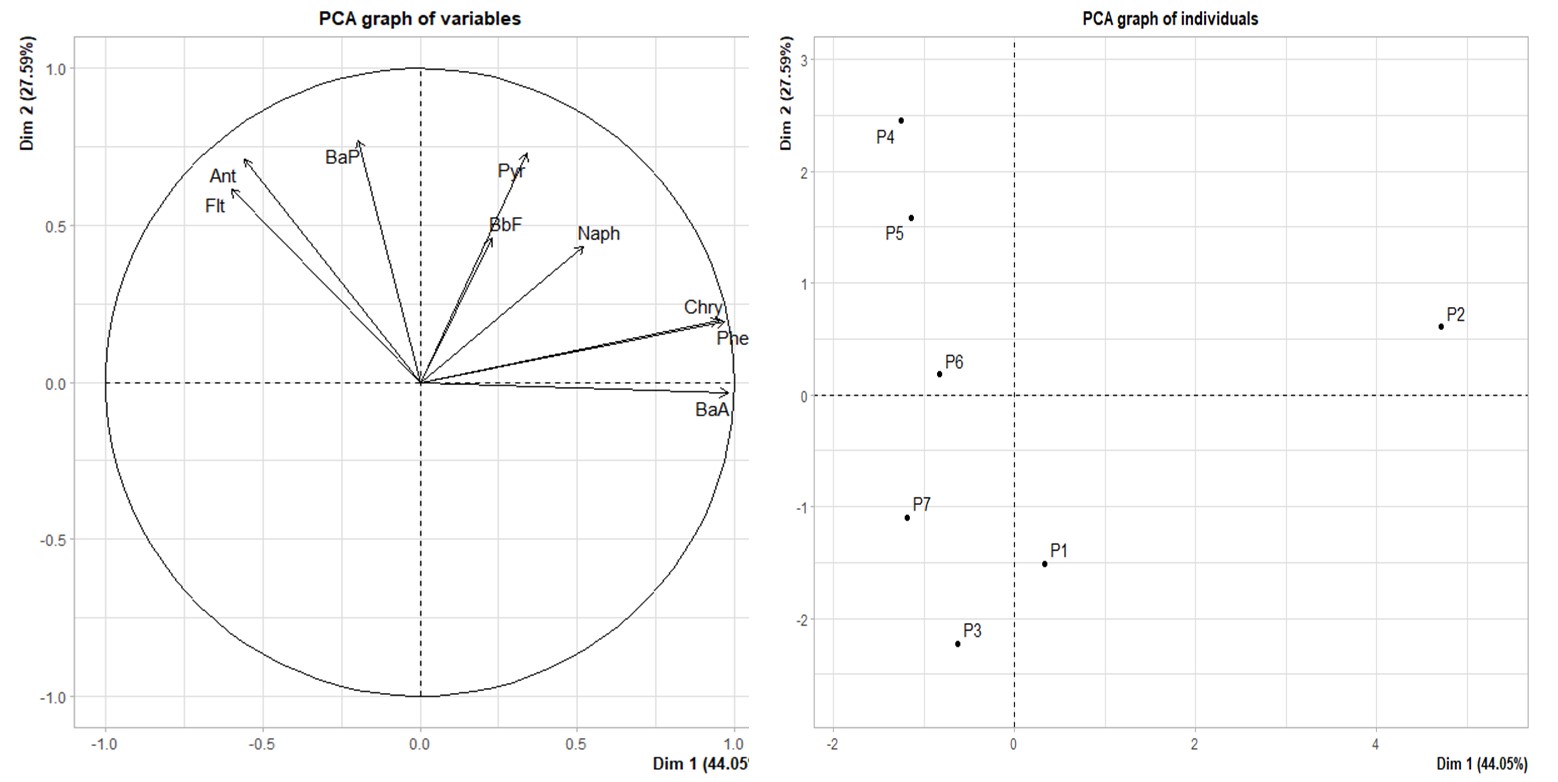


Fig. S4. Principal component analysis based on the two principal components of the data from *Acacia melanoxylon* bark.
